# Hepatic artery pseudoaneurysm caused by chronic pancreatitis: Case report and literature review

**DOI:** 10.1097/MD.0000000000032834

**Published:** 2023-02-03

**Authors:** Fengjuan Jia, Guodong Xia, Qingliang Zhu, Shuangyu Yu, Nan Hu, Hailong Zhang

**Affiliations:** a Department of Gastroenterology, The Affiliated Hospital of Southwest Medical University, Luzhou, Sichuan Province, China; b Health Management Centre, The Affiliated Hospital of Southwest Medical University, Luzhou, Sichuan Province, China.

**Keywords:** chronic pancreatitis, hepatic artery pseudoaneurysm, transarterial embolization

## Abstract

Visceral artery pseudoaneurysm is a rare complication of chronic pancreatitis (CP), all pancreatic or peripancreatic arteries have been reported to be involved, while hepatic artery is less common.

**Patient concerns:**

This case report illustrated a 42-year-old man with CP who developed right hepatic artery pseudoaneurysm (HAP), and finally he was treated with intravascular embolization.

**Diagnoses:**

The patient suffered from HAP due to acute attack of CP.

**Interventions:**

The pseudoaneurysm located in a fine branch of right hepatic artery was embolized.

**Outcomes:**

The HAP of the patient was cured. He had no recurrent bloody stool or abdominal pain. The symptoms gradually relieved.

**Conclusion:**

Herein, we report a patient with CP who developed right HAP causing infected hematoma, gastrointestinal bleeding, and obstructive jaundice, and a literature review is also presented. HAP caused by CP is a rare disease in the clinic, but rupture of pseudoaneurysm is fatal. Careful evaluation, early detection, and prompt treatment should be performed when the patient is admitted and followed up.

## 1. Introduction

Visceral artery pseudoaneurysm is an infrequent but life-threatening complication of chronic pancreatitis (CP). The most commonly involved artery is splenic artery, followed by the gastroduodenal artery, while hepatic artery is less common.^[1]^ Little is known about clinical manifestation and therapeutic strategy for hepatic artery pseudoaneurysm (HAP) in CP, and so far there are no literature reviews on this rare complication. Hence, we report a case of CP that developed HAP causing infected hematoma, gastrointestinal bleeding, and obstructive jaundice, which was successfully treated by transarterial embolization, and a literature review is also presented.

## 2. Case report

**Figure 1.**
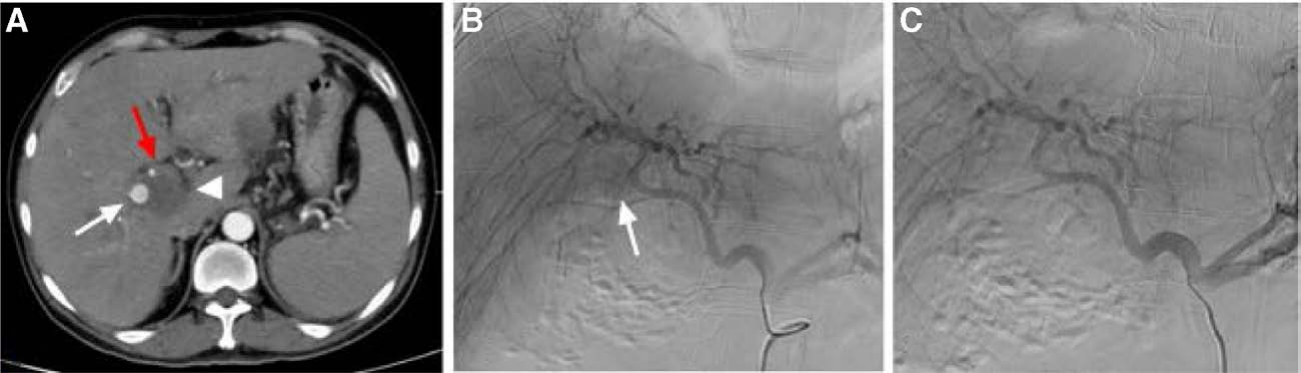
(A) Abdominal CT scan showed the presence of a right HAP (white arrow), a hematoma locating in hepatic hilum (white arrowhead) and slightly dilated bile duct (red arrow). (B)Celiac angiography confirmed the right HAP (white arrow) in the right hepatic artery, but the feeding branch of the right hepatic artery was not clear. (C) Another celiac angiography showed spontaneous disappearance of the right HAP. CT = computed tomography, HAP = hepatic artery pseudoaneurysm.

A 42-year-old man was referred to our unit because of the aggravation of hematochezia and upper abdominal pain after symptomatic treatment and blood transfusion. He had a history of CP with a pseudocyst for 4 years and drinking for 20 years without abstinence. Upon physical examination, his vital signs were stable, and the abdomen was distended with tenderness. Routine blood test showed white blood cell (WBC) count 30.94 × 10^9^/L, hemoglobin 108g/L, platelet count 436 × 10^9^/L, alanine transaminase 78.9 U/L, aspartate transaminase 126.4 U/L, total bilirubin 90.6 umol/L, direct bilirubin 72.8 umol/L, amylase, and lipase were not elevated. Initial endoscopy revealed massive blood in the stomach and a huge blood clot in duodenal bulb, but the exact bleeding source was not found. Enhanced computed tomography (CT) showed a large retroperitoneal hematoma, a right HAP, and a slightly dilated intrahepatic bile duct (Fig. 1).

**Figure 2.**
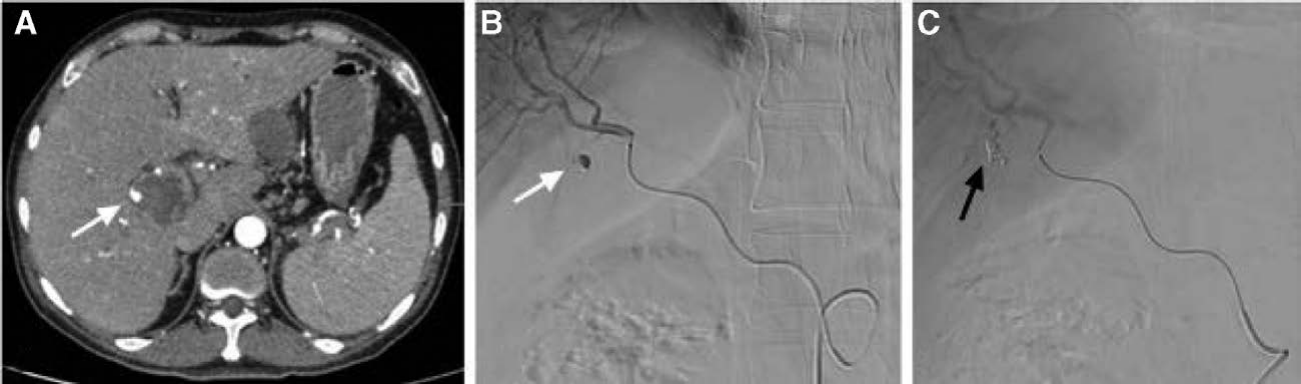
(A) Abdominal CT scan showed the presence of a right HAP with smaller diameter (white arrow), (B) Celiac angiography showed the right HAP (white arrow) in one fine branch of right hepatic artery. (C) Post embolization angiography showed occlusion of the feeding artery and pseudoaneurysm with NBCA (black arrow). CT = computed tomography, HAP = hepatic artery pseudoaneurysm, NBCA = n-butyl-2-cyanoacrylate.

**Figure 3.**
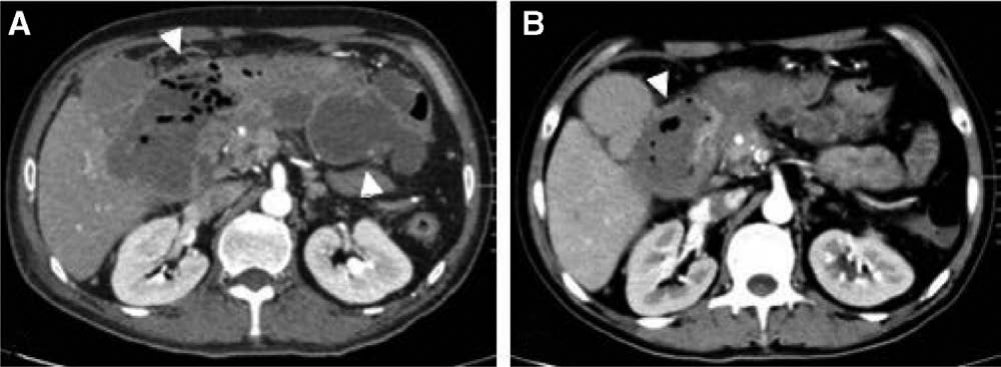
(A)Abdominal CT scan showed the presence of irregular encapsulated retroperitoneal infection containing much gas (white arrowhead). (B) CT scan 6 days after embolization showed significant regression in the size of the retroperitoneal infection (white arrowhead). CT = computed tomography.

Subsequent celiac angiography confirmed a pseudoaneurysm originating from a branch of right hepatic artery. However, the pseudoaneurysm disappeared when we selectively catheterized the right hepatic artery, and another celiac angiography showed no pseudoaneurysm. Repeated enhanced CT showed that the pseudoaneurysm still exist with smaller diameter than before the next day. So angiography was scheduled again and found the pseudoaneurysm was located in a fine branch of right hepatic artery. Micro catheter was advanced into the branch, and a mixture of n-butyl-2-cyanoacrylate and iodized oil with a ratio of 1:3 was injected successfully for embolization. After the embolization, no filling of the pseudoaneurysm could be seen (Fig. 2). Repeated endoscopy showed duodenal fistula in the duodenum bulb, but he refused jejunal nutrition tube for enteral nutrition. The clinical outcome was good. His symptoms were gradually relieved. Subsequent CT scan 6 days after the embolization showed no filling of the pseudoaneurysm with significant resolution of retroperitoneal hematoma (Fig. 3), and he was discharged 2 days later. At the time of writing this manuscript, the patient had completed 3 months of follow-up and had no recurrent hematochezia and abdominal pain.

## 3. Discussion

VPA is a rare complication of CP resulting from the erosion of pancreatic or peripancreatic vessels by leaked pancreatic enzymes or severe inflammation in patients with acute CP or communication of a peripancreatic vessel with an established pseudocyst and pseudocyst erosion.^[2]^ While we searched PubMed from January 1980 to December 2022 using the keywords “pseudoaneurysm” and “chronic pancreatitis,” only 12 cases of HAP caused by CP are reported in English literature (Table 1). Their location, symptoms, treatment, and prognosis are summarized in the table (Table 2).

**Table 1:** Summary of reported cases of HAP caused by CP.

Year	Author/Reference	Number of cases	Etiology of CP	Imaging	Angiography
2018	Zabicki^[3]^	3	No mention	CT	Yes
2017	Gabrielli^[4]^	2	No mention	Enhanced CT	Yes
2013	Gupta^[5]^	1	No mention	Enhanced CT	No
2012	Janarthanan^[6]^	1	No mention	Enhanced CT	Yes
2012	Shiraishi^[7]^	1	Alcohol	Enhanced CT	No
2007	Garg^[8]^	1	Alcohol	US/Enhanced CT	Yes
2006	Singh^[9]^	1	Alcohol	Enhanced MRI	Yes
1997	Gambiez^[10]^	1	No mention	Enhanced CT	Yes
1994	Fava^[11]^	1	No mention	US/Enhanced CT	Yes

CP = chronic pancreatitis, CT = computed tomography, HAP = hepatic artery pseudoaneurysm, MRI = magnetic resonance imaging.

**Table 2:** Details of patients with HAP.

Location	Number of cases	Symptom	Treatment	Prognosis
Common hepatic artery	10	3 with sudden upper abdominal pain	7 with transarterial embolization, 1 with percutaneous embolization, 2 with surgery.	8 were good, 1 died of bleeding†, 1 died of MODS‡.
		1 with sudden upper abdominal pain and gastrointestinal bleeding		
		1 with gastrointestinal bleeding		
		1 with obstructive jaundice		
		1 with obstructive jaundice and gastrointestinal bleeding		
		2*		
Proper hepatic artery	1	Sudden upper abdominal pain	Stenting	Good
Right hepatic artery	1	Sudden upper abdominal pain	Embolization	Good

HAP = hepatic artery pseudoaneurysm, MODS = multiple organ dysfunction syndrome.

* The symptoms of other 2 patients were not mentioned.

† The patient received percutaneous embolization.

‡ The patient received surgery.

The prevalence of pseudoaneurysm in CP has been reported as 4 to 10%,^[12,13]^ but the prevalence of HAP in CP is not clear because of its sporadical reports. In a series consisting of 647 patients with pancreatitis, only 1 patient (1.5%) had HAP.^[14]^ However, the incidence may be underestimated, because enhanced scan is not routinely performed in CP, and some patients are asymptomatic. The risk factors affecting HAP formation are also not clear, but risk factors affecting VPA formation may affect HAP formation. Alcoholic CP was the most significant predictor for the development of pseudoaneurysm, followed by the presence of pseudocyst.^[15]^

Review of the literature revealed that the incidence of common hepatic artery injury is much higher than proper hepatic artery and right or left hepatic artery. Generally, the artery is closer to the pancreas, the arterial injury is more likely to occur. In our case, we presumed that HAP was caused by pseudocyst erosion because his amylase and lipase were not elevated and CT scan did not show signs of acute on CP.

Similar to other VPA, symptoms of HAP not only depend on its size and location but also on whether it ruptures. Except for decrease in hemoglobin, there are some other symptoms that may be a clue to suspect pseudoaneurysm rupture occurring. The peritoneal and retroperitoneal bleeding or hematoma can present upper abdominal pain exacerbation.^[8,9]^ Hematoma compressing bile duct may cause jaundice.^[7]^ Hematoma communicating with gastrointestinal track can cause gastrointestinal bleeding.^[11]^ Our case is the first report of coexistence of gastrointestinal bleeding and obstructive jaundice, meanwhile, due to susceptibility to infection and duodenal fistula, retroperitoneal infection also existed in our patient. Initial antibiotic therapy seemed to be no effect with no resolve in upper abdominal pain and progressive elevation of WBC count. However, after embolization, retroperitoneal infection was found surprisingly relieved with smaller size and normalization in WBC count.

When suspecting pseudoaneurysm occurring, early diagnosis and treatment are critical to prognosis, because life-threatening complications may occur. As demonstrated by table, ultrasound and enhanced CT or magnetic resonance imaging (MRI) play the important role in detecting HAP. Subsequent angiography is the golden criterion of pseudoaneurysm and should be done as soon as possible when suspected by ultrasound and enhanced CT or MRI.

Conservative management, including hemostasis, transfusion, and antibiotics if infection happens, should be given. Although Yu reported a pseudoaneurysm spontaneously thrombosed without any particular treatment,^[16]^ our case also showed a tendency to resolve spontaneously because of disappearance in angiography procedure and smaller cavities detected by CT the next day. More aggressive treatment, including surgery, endovascular treatment should be given in most cases. Endovascular treatment is the first choice recommended by most studies, but preprocedure imaging and angiography are integral to choosing appropriate endovascular treatment or surgery. In a case reported by Gupta, endovascular access to the HAP was infeasible due to the coexistent celiac axis stenosis and complex collaterals.^[5]^ In another case reported by Shiraishi, surgery was planned because of difficulty in embolization of the pseudoaneurysm cavity, in addition to the need for surgical release of the common bile duct obstruction.^[7]^ Endovascular treatment or surgery depends on the location of pseudoaneurysm, operator experience, and multidisciplinary discussion is essential for selected patients, including those with large pseudoaneurysms, complex collaterals, or special locations.

## 4. Conclusion

HAP is a rare but potentially life-threatening complication. Its occurrence should be considered in any patients with upper abdominal pain exacerbation, gastrointestinal bleeding, obstructive jaundice, or peritoneal and retroperitoneal bleeding. Ultrasound and enhanced CT or MRI is the diagnostic modality of choice, angiography is the golden criterion of pseudoaneurysm. Preprocedure imaging and angiography are integral to choosing appropriate endovascular treatment or surgery.

## Author contributions

**Data curation:** Fengjuan Jia, Qingliang Zhu, Hailong Zhang.

**Formal analysis:** Fengjuan Jia, Shuangyu Yu.

**Investigation:** Fengjuan Jia, Guodong Xia, Nan Hu.

**Methodology:** Fengjuan Jia, Shuangyu Yu, Nan Hu.

**Resources:** Fengjuan Jia, Qingliang Zhu, Hailong Zhang.

**Supervision:** Fengjuan Jia, Guodong Xia.

**Validation:** Guodong Xia, Shuangyu Yu, Hailong Zhang.

**Writing – original draft:** Fengjuan Jia.

**Writing – review & editing:** Fengjuan Jia, Guodong Xia, Nan Hu.
